# The Diagnostic and Prognostic Potentials of Non-Coding RNA in Cholangiocarcinoma

**DOI:** 10.3390/ijms25116002

**Published:** 2024-05-30

**Authors:** Rita Andrade, Ilda Patrícia Ribeiro, Isabel Marques Carreira, José Guilherme Tralhão

**Affiliations:** 1 Surgery Department, Centro Hospitalar e Universitario de Coimbra EPE (CHUC), 3000-075 Coimbra, Portugal; ritaandrade03@gmail.com; 2 Clinical Academic Center of Coimbra, Faculty of Medicine, University of Coimbra, 3000-548 Coimbra, Portugal; 3 Cytogenetics and Genomics Laboratory, Institute of Cellular and Molecular Biology, Faculty of Medicine, University of Coimbra, 3000-548 Coimbra, Portugal; 4 Coimbra Institute for Clinical and Biomedical Research (CBR) and Center of Investigation on Environment Genetics and Oncobiology (CIMAGO), Faculty of Medicine, University of Coimbra, 3000-548 Coimbra, Portugal; 5 Center for Innovative Biomedicine and Biotechnology (CIBB), University of Coimbra, 3000-548 Coimbra, Portugal

**Keywords:** cholangiocarcinoma, non-coding RNA, long non-coding RNA, circular RNA, biomarker, diagnosis, prognosis

## Abstract

Cholangiocarcinoma (CCA) is a rare biliary tract tumor with high malignancy. CCA is the second most common primary hepatobiliary cancer after hepatocarcinoma. Despite its rarity, the incidence of CCA is steadily increasing globally. Most patients with CCA are asymptomatic in the early stages, resulting in a late-stage diagnosis and poor prognosis. Finding reliable biomarkers is essential to improve CCA’s early diagnosis and survival rate. Non-coding RNAs (ncRNAs) are non-protein coding RNAs produced by genomic transcription. This includes microRNAs, long non-coding RNAs, and circular RNAs. ncRNAs have multiple functions in regulating gene expression and are crucial for maintaining normal cell function and developing diseases. Many studies have shown that aberrantly expressed ncRNAs can regulate the occurrence and development of CCA. ncRNAs can be easily extracted and detected through tumor tissue and liquid biopsies, representing a potential tool for diagnosing and prognosis CCA. This review will provide a detailed update on the diagnostic and prognostic potentials of lncRNAs and cirRNAs as biomarkers in CCA.

## 1. Introduction

Cholangiocarcinoma (CCA) is a highly aggressive malignancy from the epithelial cells lining the bile ducts [1]. While CCA is relatively rare, comprising less than 2% of all human malignant neoplasms, it ranks as the second most prevalent primary hepatobiliary cancer, accounting for approximately 10–25% of cases globally [2]. In recent years, epidemiological studies have noted a concerning rise in the incidence of CCA worldwide [3]. Numerous risk factors contribute to the development of CCA, including chronic infections with hepatitis B and hepatitis C viruses, tobacco smoking, heavy alcohol consumption, obesity, and non-alcoholic fatty liver disease [4]. The multifactorial nature of CCA underscores the complex interplay between genetic predisposition and environmental factors in disease pathogenesis.

It can be classified according to its anatomic origin: intrahepatic cholangiocarcinoma (iCCA), perihilar cholangiocarcinoma (pCCA), and distal cholangiocarcinoma (dCCA) [1]. iCCA can show three main patterns of growth, namely mass-forming, periductal-infiltrating, and intraductal-growing; pCCA and dCCA present as flat or poorly defined nodular sclerosing tumors or, less frequently, as intraductal papillary tumors [5]. Pre-invasive lesions can precede CCA. Although most pCCA and dCCA are conventional mucin-producing adenocarcinomas or papillary tumors, iCCA shows several histological variants (conventional, cholangiocarcinoma, and rare variants). Conventional iCCA can be further classified into two main histological subtypes according to the level or size of the affected site. Small bile duct iCCA presents as a small-sized tubular or acinar adenocarcinoma with nodular growth invading the liver parenchyma and with no or minimal mucin production. Large bile duct iCCA arises in sizeable intrahepatic bile ducts and comprises mucin-producing columnar tumor cells arranged in a large duct or papillary architecture [5].

Despite the considerable advances in the field of diagnostic imaging modalities and therapeutic interventions, the prognosis for patients with CCA remains poor. 

The most common symptom of both pCCA and dCCA is jaundice, which occurs due to the obstruction of the bile duct. However, in the case of iCCA, jaundice is less common and is usually associated with advanced disease. Other symptoms of advanced-stage liver cancer include weakness, abdominal pain, fatigue, nausea, loss of appetite, and weight loss [1,5].

Imaging techniques, such as computed tomography (CT) and magnetic resonance imaging (MRI), play a crucial part in managing CCA regarding diagnosis, staging, follow-up, and assessment of treatment response. Cholangiocarcinoma diagnosis and staging accuracy vary with anatomical location and growth patterns [1]. CT is considered the standard imaging method for preoperatively evaluating both iCCA and pCCA; it comprehensively evaluates the primary tumor, the relationship with adjacent structures, and potential thoracic and abdominal spread. MRI has similar accuracy to CT for diagnosis and staging. Still, it incorporates specific sequences such as diffusion-weighted imaging and the potential for performing magnetic resonance cholangiopancreatography (MRCP), which is critical for pCCA staging [5]. 

Confirmation of diagnosis requires histopathological or cytological analysis, as no specific CCA radiology pattern exists. This diagnosis is based on the WHO classification of biliary tract cancer. The classification shows an adenocarcinoma or mucinous carcinoma with tubular and papillary structures and a variable fibrous stroma [5].

CCA management with curative intent requires a surgical approach, but the 5-year survival rate ranged from a modest 20% to 40% following surgical resection [6]. Despite recent improvements in medicine, due to the lack of early specific clinical symptoms and the tumor’s rapid growth, most cases are detected at an advanced stage. Consequently, they are not candidates for curative treatment [5]. In non-surgical patients, gemcitabine in combination with cisplatin is considered the standard chemotherapy regimen. However, they have poor chemoresistance and limited efficiency, with a median survival of only 11.7 months [7]. 

Exploring and understanding the molecular pathogenesis of CCA to find sensitive and specific biomarkers is crucial to improving the early diagnosis and survival rate. 

**Figure 1:**
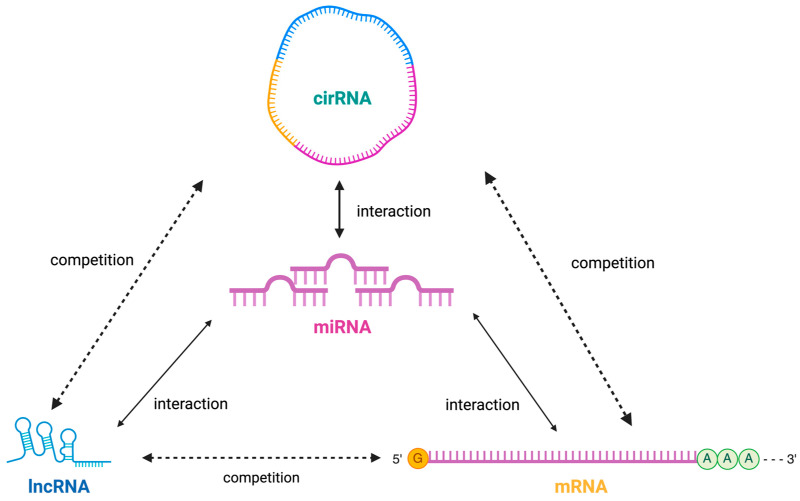
Interaction of different ncRNAs (miRNAs, lncRNAs, and circRNAs): these three non-coding RNAs may interact and function independently.

Recent advancements in molecular biology have shed light on the role of non-coding RNAs (ncRNAs) in cancer development and progression, including their potential as diagnostic and prognostic biomarkers in CCA. Non-coding RNAs (ncRNAs) are a heterogeneous class of RNA molecules without protein-coding ability, including microRNAs (miRNAs), long non-coding RNAs (lncRNAs), and circular RNAs (circRNAs), which account for 98% of the human genome [8]. These three non-coding RNAs may interact and function independently (Figure 1). ncRNAs can act as regulatory molecules that mediate cellular processes such as chromatin remodeling, transcription, post-transcriptional changes, and signal transduction [9]. In recent years, studies have found that ncRNAs can participate in the biological processes of cancer as oncogenes or suppressors and play a critical role in regulating the progression of various cancers [10]. A growing number of studies have shown that abnormally expressed ncRNAs can regulate the occurrence and development of CCA [11,12]. ncRNAs can be easily extracted and detected through tumor tissue and liquid biopsies from blood, urine, and other bodily fluids, representing a potential tool for diagnosing and prognosis of CCA.

The most widely studied class of ncRNAs is the miRNAs, and reviews of their molecular roles in CCA have been extensively reported [13]. Therefore, this review article will emphasize lncRNAs and circRNAs. 

This paper will provide a retrospective view of the latest research on the link between these molecules and CCA, discussing the diagnostic and prognostic potentials of lncRNAs and cirRNAs as biomarkers for CCA in clinical practice. By elucidating the molecular mechanisms underlying CCA development and progression, we aim to underscore the importance of ncRNAs as promising targets for early detection and improved patient outcomes.

## 2. lncRNAs as Diagnostic and Prognostic Biomarkers in Cholangiocarcinoma 

lncRNAs represent non-coding RNAs longer than 200 nucleotides, and recent reports demonstrated that they are involved in carcinogenesis or tumor suppression [14]. lncRNAs are mainly formed by RNA polymerase II transcription and are widely distributed in the nucleus and cytoplasm. Like mRNAs, lncRNAs have a 5’-cap structure and a 3’-end nucleotide polymer tail structure that enables gene splicing, but they lack a complete reading frame, and thus, lncRNAs themselves do not encode a functional protein [15]. According to their association with mRNAs, lncRNAs can be categorized into five types: (1) sense, (2) antisense, (3) intronic, (4) intergenic, and (5) bidirectional [16]. lncRNAs participate in diverse biological processes involving chromatin modification, gene transcription, and translation. They have extensive roles according to their cellular location: In the nucleus, the functions mainly comprise chromatin interaction, RNA processing, and transcriptional regulation, whereas lncRNAs in the cytoplasm can interact with miRNAs, functioning as competitive endogenous RNAs (ceRNAs) to modulate mRNAs and cellular signaling pathways [17]. 

Many abnormally expressed lncRNAs have been discovered between CCA and adjacent normal tissues [18]. Numerous lncRNAs localized in exosomes can be secreted into bodily fluids (plasma, urine, and bile) as circulating RNAs with high tissue specificity, becoming potential non-invasive biomarkers [19]. 

In recent research, a few lncRNAs were identified in serum and urine extracellular vesicles (EVs) from patients with CCA, primary sclerosing cholangitis (PSC), and healthy individuals. In serum EVs, *MALAT1* and *LOC100190986* were highly accurate for differentiating CCA and PSC, with an area under the curve (AUC) value of 1.0. *LOC100134868*, while they exhibited a diagnostic value in urine EVs with an AUC of 0.896 (CCA vs. healthy controls) [19]. 

*MALAT1* was established to be upregulated in various tumor tissues, and the overexpression of *MALAT1* was associated with adverse clinical features. The overexpression of *MALAT1* is correlated with a lower overall survival (OS) rate, worse tumor node metastasis (TNM) stage, larger tumor size, and metastasis in pCCA patients. Shi et al. showed that *MALAT1* in plasma has specificity and sensitivity as a diagnostic biomarker of pCCA (AUC, 0.860; sensitivity, 81.1%; specificity, 90.9%). Further, when *PCAT1*, MALAT1, and *CPS1-IT1* were combined, the sensitivity and specificity increased to 85.5% and 93.2%, respectively [20].

A study found that *PCAT1* is elevated in extrahepatic cholangiocarcinoma and can regulate their progression through the Wnt/β-catenin signaling pathway [21]. An updated analysis showed that YY1-induced lncRNA *PCAT1* supported the progression of CCA through the miR-216a-3p/BCL3 axis [22]. The overexpressed *PCAT1* was established as a factor associated with the adverse outcome for CCA patients and made an effective prognostic prediction with an AUC of 0.823. Regarding its diagnostic potential, the AUC of plasma *PCAT1* was 0.784 based on ROC (receiver operating characteristic) curves [20]. 

Ge et al. observed that two exosomal lncRNAs, *ENST00000588480.1* and *ENST00000517758.1*, isolated and identified from human bile samples, were highly expressed in CCA patients. When these two lncRNAs were combined for diagnosis, their AUC, sensitivity, and specificity were 0.709, 82.9%, and 58.9%, respectively. Their sensitivity was superior to that of serum CA-19.9 (82.9% vs. 74.3%). In this study, the authors also confirmed that the higher the expression of these two lncRNAs in CCA patients, the worse their survival [23]. 

In iCCA, a study reported that *lncRNA-NEF* was downregulated in the tumor tissues, and upregulated *lncRNA-NEF* expression repressed cell migration and invasion by inhibiting runt-related transcription factor 1 (*RUNX1*). The level of plasma *lncRNA-NEF* in patients was significantly lower than in healthy controls and exhibited the ability for early diagnosis with an AUC of 0.8642. Nevertheless, this study did not mention the sensitivity and specificity of *lncRNA-NEF* as a diagnostic marker for iCCA. OS was significantly lower in iCCA patients with low *lncRNA-NEF* expression (*p* = 0.0198) [24]. 

It was found that *DLEU1* was associated with advanced TNM stage and lymph node infiltration, and CCA tumors with high *DLEU1* expression had poorer OS. *DLEU1* showed an AUC, sensitivity, and specificity of 0.747, 72.4%, and 65.4%, respectively [25].

Studies have shown that *H19* is upregulated in CCA tissues, which is associated with tumor size, TNM stage, postoperative recurrence, and overall survival [26]. *H19* was observed to have moderate sensitivity in distinguishing CCA tissues from normal ones, with an AUC of 0.7422. Moreover, a combination of *AC005550.3*, *H19*, *C3P1*, and *PVT1*, along with *LPAL2*, showed higher sensitivity (93.75%) and specificity (81.25%), with an AUC of 0.8828, in differentiating CCA tissues from controls [27]. 

Jiang et al. observed that the expression level of *CCAT1* in CCA tumor tissues was significantly higher than that in paired adjacent normal tissues, and the upregulated expression of *CCAT1* was associated with poor histological differentiation, lymph node invasion, and advanced TNM stage. The OS rate of lncRNA *CCAT1*-overexpressed patients was significantly lower than underexpressed patients, and by using multivariate and ROC analysis (sensitivity: 81.8%, specificity: 74.5%), lncRNA *CCAT1* was found to be an independent prognostic factor for CCA [28]. 

*CCAT2* expression is upregulated in CCA, and this high expression may be correlated with CCA progression and metastasis. The level of *CCAT2* expression is inversely related to the overall survival rate of CCA patients. Recent evidence suggests that *CCAT2* has practical value in predicting the prognosis of patients with this tumor, with an AUC of 0.702 and 0.715 for OS and PFS, respectively [29]. 

It was proved that *ZEB1-AS1* was overexpressed in CCA and promoted CCA growth, along with metastasis in in vivo and in vitro experiments. A higher *ZEB1-AS1* expression was associated with lymph node invasion, progressed TNM stage, and shorter survival time. Concerning prognostic efficiency, the AUC of *ZEB1- AS1* was 0.749 with 65.5% sensitivity and 80.0% specificity in ROC analysis [30].

To shed light on the role of lncRNAs as biomarkers in cholangiocarcinoma, Table 1 presents a detailed summary of recent studies demonstrating their diagnostic and prognostic significance.

## 3. circRNAs as Diagnostic and Prognostic Biomarkers in Cholangiocarcinoma 

circRNAs are covalently closed single-stranded RNAs lacking 5′ m7G caps or 3′ poly(A) tails and are produced from the intermediate exons of protein-coding genes [31]. Studies have indicated that circRNAs have vital functions such as modulating transcription, regulating alternative splicing, sponging for proteins and miRNAs, forming functional circRNP complexes, interacting with messenger RNAs to affect their expression, and translating into proteins [15,32]. Due to the tissue specificity and stability of circRNAs (not easily degraded by ribonuclease R), they are considered potential reliable biomarkers for cancer [33,34].

A recent study reveals that the expression of the circRNA Cdr1as was markedly elevated in CCA tissues relative to adjacent normal tissues and was highly related to lymph node infiltration progressed TNM and postoperative recurrence. The overall survival of CCAA patients with high Cdr1as expression was worse than that of the CCA patients with low Cdr1as expression. Cdr1as could be considered an independent prognostic marker to predict overall survival in CCA patients (sensitivity = 83.3%, specificity = 58.3%) [35]. 

Xu et al. found upregulated hsa_circ_102066 (*circ-CCAC1*) levels in bile and serum-derived EVs of CCA patients, which have good diagnostic properties. The diagnostic value of serum EVs (AUC = 0.759) was almost identical to that of serum CA-19.9 (AUC = 0.757), but bile EVs (0.857) were superior to CA-19.9. Interestingly, the combination of both bile- or serum-derived EV-circ-CCAC1 and serum CA-19.9 had better diagnostic performance than alone. This study established that high expression (*p* = 0.001) of *circ-CCAC1* was an independent prognostic biomarker for iCCA and that *circ-CCAC1* expression predicted the postoperative recurrence of iCCA (*p* = 0.002) [36].

It was reported that hsa_circ_0000284 (cir-HIPK3) was increased in plasma exosomes of CCA patients [37]. This study suggests that *circ-0000284* may transfer from cholangiocarcinoma cells to surrounding normal cells through exosomes and modulate the biological functions of surrounding normal cells. Another study observed that hsa_circ_0020256 (*cir-NSMCE4A*) was highly expressed in exosomes secreted by tumor-associated macrophages, which promoted proliferation, migration, and invasion of CCA cells [38]. This study also demonstrated that time to relapse and OS in CCA patients were negatively correlated with hsa_circ_0020256 expression.

Studies demonstrated that hsa_circ_0043469 (*cir-ERBB2*) was significantly upregulated in exosomes derived from the bile of CCA patients [39], and higher levels of hsa_circ_0030998 (*cir-LAMP1*) were also associated with increased postoperative recurrence rates [40]. 

Hsa_circ_0003930 (cir-GGNBP2) was expressed at much higher levels in iCCA than in matched non-tumors, and it was associated with shorter OS and recurrence-free survival (RFS) in patients after surgical resection. Cox proportional hazard regression models showed that elevated hsa_circ_0003930 was an independent risk factor for OS and RFS and could be considered a prognostic factor for iCCA [41]. 

Zhang X et al. described that hsa_circ_0059961 (*cir-ITCH*) expression was decreased in CCA tissues. In this study, the Pearson correlation analysis found that the survival time of CCA patients was positively correlated with hsa_circ_0059961 expression, and Kaplan–Meier survival analysis demonstrated that the OS rate of patients with high expression of hsa_circ_0059961 was higher than that of patients with low expression [42].

Patients with higher expression of hsa_circ_0008621 (*cir-HMGCS1*) in iCCA tissues exhibited significantly shorter survival time and higher cumulative recurrence rate after radical resection, indicating that the level hsa_circ_0008621 was an independent prognostic indicator for ICCA patients’ cumulative recurrence [43].

To further elucidate the role of circRNAs as biomarkers in cholangiocarcinoma, Table 2 provides a comprehensive overview of recent studies highlighting their diagnostic and prognostic potential.

## 4. Discussion

Cholangiocarcinoma (CCA) is a rare but aggressive malignancy arising from the bile duct epithelium, characterized by its high mortality rate and limited treatment options. While it accounts for less than 2% of all human cancers, CCA ranks as the second most common primary hepatobiliary cancer globally, posing significant challenges to clinical management. The classification of CCA is based on its anatomical location, with intrahepatic (iCCA), perihilar (pCCA), and distal (dCCA) subtypes exhibiting distinct clinical and molecular features. Despite advancements in diagnostic imaging and therapeutic modalities, the prognosis for CCA remains dismal, with most patients presenting at advanced stages and facing limited treatment options.

Epidemiological studies have reported a rising incidence of CCA worldwide, attributed to various risk factors, including chronic liver diseases, biliary tract disorders, and environmental exposures. Hepatitis B and C virus infections, chronic inflammation, bile duct cysts, and primary sclerosing cholangitis are established predisposing factors in CCA development. Moreover, lifestyle factors such as smoking, obesity, and heavy alcohol consumption have also been associated with an increased risk of CCA, highlighting the multifactorial etiology of this disease.

Despite advances in surgical techniques and perioperative care, the prognosis for patients with CCA remains poor, with a five-year survival rate ranging from 10% to 30%. Surgical resection with negative margins represents the only potentially curative treatment option for localized disease; however, only a minority of patients are deemed suitable candidates for curative-intent surgery due to late-stage presentation and underlying comorbidities. Furthermore, the high rate of disease recurrence following surgical resection underscores the need for effective adjuvant therapies and reliable biomarkers for prognostic stratification. 

The diagnosis of CCA poses significant challenges due to its non-specific clinical presentation and overlapping radiological features with other hepatobiliary disorders. While imaging modalities such as CT and MRI are crucial in detecting and characterizing biliary lesions, histopathological confirmation remains essential for definitive diagnosis. Percutaneous liver biopsy, although invasive, remains the gold standard for tissue sampling and histological analysis; however, it carries inherent risks of complications such as hemorrhage and tumor seeding [63]. New biomarkers are needed to improve early-stage diagnosis, predict prognosis, and monitor recurrence. Unfortunately, there is no effective and reliable biomarker for the diagnosis or prognosis of cholangiocarcinoma. The most used biomarkers are CA-19.9 and CEA in diagnosing CCA, whether alone or in combination. Still, their value is limited due to the inconsistency in sensitivity (47.2–98.2%) and specificity (89.7–100%) [64,65].

CCA carcinogenesis results from several oncogenes, tumor suppressor genes, and associated molecular disorders. Revealing the molecular mechanisms underlying this tumor may contribute to finding reliable biomarkers. 

In recent years, many studies have discovered that ncRNAs play an essential role in the occurrence and progression of CCA. These RNA molecules lack protein-coding capacity and play diverse regulatory roles in gene expression and cellular processes, making them attractive candidates for biomarker discovery. With the developments in high-throughput sequencing and molecular gene technologies, it has been demonstrated that these molecules are abnormally expressed in the tissues, cells, and bile of CCA. 

Among the various classes of ncRNAs, microRNAs (miRNAs), long non-coding RNAs (lncRNAs), and circular RNAs (circRNAs) have emerged as critical regulators of tumorigenesis and disease progression in CCA. miRNAs are small, endogenous ncRNAs that modulate gene expression post-transcriptionally by binding to the 3’ untranslated region (UTR) of target mRNAs, leading to translational repression or mRNA degradation. Dysregulated miRNA expression has been implicated in CCA pathogenesis, with specific miRNAs acting as oncogenes or tumor suppressors. For example, miR-21, miR-31, and miR-155 are upregulated in CCA tissues and promote tumor growth, invasion, and metastasis by targeting tumor suppressor genes involved in cell cycle regulation and apoptosis [10].

In addition to miRNAs, lncRNAs represent another class implicated in CCA development and progression. lncRNAs are structurally like mRNAs but lack protein-coding capacity, enabling them to regulate gene expression at the transcriptional and post-transcriptional levels. Emerging evidence suggests that dysregulated lncRNA expression contributes to CCA pathogenesis through various mechanisms, including chromatin remodeling, transcriptional regulation, and modulation of signaling pathways. 

circRNAs represent a distinct class of ncRNAs characterized by covalently closed loop structures formed by back-splicing exons or introns. circRNAs exhibit remarkable stability and resistance to exonuclease-mediated degradation, rendering them attractive candidates as diagnostic and prognostic biomarkers. Evidence suggests that dysregulated circRNA expression is associated with CCA development and progression, with specific circRNAs acting as oncogenes or tumor suppressors.

By monitoring ncRNAs in biofluids, we are taking a crucial step toward improving cancer research in CCA. They have diagnostic and prognostic potential as CCA biomarkers due to their extensive distribution and relatively stable structure. Analyzing ncRNAs in biofluids is less invasive than traditional tissue biopsies, allowing for repeated sampling and ongoing disease progression and treatment response monitoring. CCA exhibits significant heterogeneity among patients, and personalized treatment demands accurate characterization. The non-invasive profiling of ncRNAs in biofluids provides a promising approach to capture this heterogeneity and enable personalized medicine.

While the discussion on the extraction and detection of ncRNAs provides valuable insights, it is essential to acknowledge the importance of detailed methodological approaches for reproducibility and validation of results, particularly in a clinical setting. Future studies should strive to incorporate robust methodologies, including standardized protocols for sample collection, RNA extraction, and detection techniques, to ensure the reliability and validity of findings. Moreover, the establishment of quality control measures and the inclusion of appropriate controls are imperative steps to enhance the clinical utility of ncRNAs as biomarkers.

This review revealed that this abnormal expression is remarkably linked to adverse clinical features and OS, which means that ncRNAs may be used as effective indicators for risk stratification and predicting survival in CCA patients.

Despite promising potential as biomarkers, ncRNAs face translational barriers in clinical practice for CCA and other cancers. Many studies on ncRNAs were conducted with relatively small cohorts, which can lead to false-positive results and limited generalizability. Some studies need more reproducibility, and validation studies are critical for confirming the clinical utility of ncRNA biomarkers. The lack of rigorous validation studies in large and diverse patient cohorts has hindered the translation of ncRNAs into clinical practice. To ensure reliable and comparable ncRNA data across different research groups, it is essential to standardize methodologies for sample collection, RNA extraction, sequencing, and data analysis. Despite these challenges, it is important to note that the field of ncRNAs is still relatively young, and ongoing research seeks to address these issues. 

Other biomarkers are currently being researched for their potential in diagnosing and predicting outcomes in cholangiocarcinoma. These include microRNAs and exosomes. miR-21 has been associated with tumor progression and poor prognosis, while miR-34a and miR-122 have been linked to tumor suppression and better clinical outcomes [13]. Recent research has shown that miR-150 and miR-200c could be used as potential diagnostic biomarkers, differentiating cholangiocarcinoma from other liver disorders with high sensitivity and specificity [13]. Additionally, exosomes, which are small extracellular vesicles, carry molecular cargoes such as proteins, lipids, and RNAs that reflect the physiological state of their cells of origin [5]. Exosomal miRNAs have been found to differentiate between cholangiocarcinoma and benign biliary diseases, offering a non-invasive diagnostic tool. For example, exosomal miR-21 and miR-1246 have been identified as potential biomarkers for the early detection of cholangiocarcinoma [13].

Although there has been a growing number of studies and encouraging results, more research is still required to demonstrate the value of ncRNAs in the clinical management of patients with CCA. As technology advances and more data become available, some ncRNAs may eventually find their way into clinical practice. The scientific community is making progress in improving the reproducibility of findings, validating potential biomarkers, and developing standardized methodologies. However, translating ncRNAs into clinical practice can be time-consuming and resource-intensive, requiring researchers and clinicians to collaborate. With continued research efforts, ncRNAs may eventually become a valuable tool in the clinical management of CCA patients, but it will take time, resources, and collaboration to achieve this goal.

## 5. Conclusions

nlRNAs, such as lnRNAs and circRNAs, are emerging as potential tumor diagnostic and prognostic biomarkers and can be incorporated into the management of CCA, improving the survival rate of CCA patients. Despite the complexities and challenges associated with their clinical translation, ongoing research efforts aimed at elucidating the functional roles of ncRNAs in CCA pathogenesis and validating their clinical utility hold the potential to revolutionize the management of this devastating disease. However, more investigation, particularly through large-scale, multi-center studies, is imperative to translate ncRNAs into clinical use and validate their utility as biomarkers.

## Figures and Tables

**Table 1:** lncRNAs as biomarkers of CCA.

LncRNA	Sample	Expression	Clinical Application	OS	AUC	miRNA Interactions	Ref.
ANRIL	Tissue	Up	Tumor size	√	-	miR-99a, miR-125a	[31]
ASAP1-IT1	Tissue	Up	Lymph node invasion, TNM stage, postoperative recurrence	√	-	miR-30c, miR-124	[15]
CCAT1	Tissue	Up	Lymph node invasion, TNM stage	√	0.831	miR-155, miR-143	[28]
CCAT2	Tissue	Up	Lymph node invasion, TNM stage, microvascular invasion	√	0.702	miR-145, miR-143	[29]
CRNDE	Tissue	Up	Poor differentiation, tumor size, lymph node invasion, TNM stage	√	-	miR-136, miR-217	[32]
DLEU1	Tissue	Up	Lymph node invasion, TNM stage	√	0.747	miR-22, miR-181a	[25]
FOXD2-AS1	Tissue	Up	Lymph node invasion, TNM stage	√	0.741	miR-185, miR-124	[33]
GAPLINC	Tissue	Up	Lymph node invasion, TNM stage	√	0.713	miR-211, miR-29b	[34]
H19	Tissue	Up	Tumor size, TNM stage, postoperative recurrence	√	-	miR-675, miR-200	[26]
HOTAIR	Tissue	Up	Lymph node invasion, TNM stage, postoperative recurrence	√	-	miR-141, miR-23b	[35]
HOTTIP	Tissue	Up	Lymph node invasion, distant metastasis	√	-	miR-615, miR-148a	[36]
HOXD-AS1	Tissue	Up	Lymph node invasion, TNM stage	√	0.786	miR-125b, miR-130a	[37]
LINC00261	Tissue	Up	Tumor size, lymph node invasion, TNM stage, postoperative recurrence	√	-	miR-588, miR-34a	[38]
LINC00665	Tissue	Up	Lymph node invasion, distant metastasis, TNM stage	√	-	miR-138, miR-3194	[39]
LINC00667	Tissue	Up	Lymph node invasion, TNM stage	√	0.830	miR-361, miR-129	[40]
LINC01296	Tissue	Up	Lymph node invasion, TNM stage	√	-	miR-26a, miR-98	[41]
lncRNA-NEF	Plasma	Down	Diagnostic marker, lymph node invasion, TNM stage	√	0.864	miR-155, miR-497	[24]
LOXL1-AS1	Tissue	Up	Lymph node invasion, TNM stage	√	-	miR-589, miR-23a	[42]
MALAT1	Tissue, plasma	Up	Tumor size, pathological T stage, perineural invasion	√	-	miR-145, miR-124	[20]
NNT-AS1	Tissue	Up	Tumor size, lymph node invasion, TNM stage	√	-	miR-483, miR-223	[43]
PAICC	Tissue	Up	Tumor number, tumor size, vascular invasion	√	-	miR-125b, miR-34c	[44]
PANDAR	Tissue	Up	Lymph node invasion, TNM stage, postoperative recurrence	√	-	miR-150, miR-10b	[45]
PCAT1	Tissue	Up	Lymph node invasion, TNM stage	√	0.823	miR-203, miR-21	[21,46]
PKD2-2-3	Tissue	Up	Poor differentiation, TNM stage	√	-	miR-125a, miR-192	[47]
PSMA3-AS1	Tissue	Up	Lymph node invasion, TNM stage	√	0.793	miR-489, miR-214	[48]
SNHG3	Tissue	Up	Lymph node invasion, distant metastasis, TNM stage	√	-	miR-338, miR-124	[49]
SNHG20	Tissue	Up	Lymph node invasion, distant metastasis, TNM stage	√	0.748	miR-140, miR-224	[50]
Sox2ot	Tissue	Up	Lymph node invasion, TNM stage, postoperative recurrence	√	-	miR-211, miR-134	[51]
TUG1	Tissue	Up	Tumor stage, intrahepatic metastasis, lymph node metastasis, perineural invasion	√	-	miR-299, miR-335	[52]
UCA1	Tissue	Up	Lymph node invasion, TNM stage, postoperative recurrence	√	-	miR-138, miR-507	[53]
ZEB1-AS1	Tissue	Up	Lymph node invasion, TNM stage	√	0.749	miR-200b, miR-141	[54]
ZFAS1	Tissue	Up	Lymph node invasion, TNM stage, postoperative recurrence	√	-	miR-150, miR-1271	[55]

**Table 2:** cirRNAs as biomarkers of CCA.

CircRNA	Sample	Expression	Clinical Application	OS	AUC	miRNA Interactions	Ref.
Cdr1as	Tissue	Up	Lymph node infiltration, progressed TNM, postoperative recurrence	√	-	miR-7	[56]
hsa_circ_102066 (circ-CCAC1)	Bile, Serum EVs	Up	Diagnostic marker, postoperative recurrence	√	0.759	miR-514a-5p, miR-320a	[57]
hsa_circ_0000284 (cir-HIPK3)	Plasma exosomes	Up	Biological functions modulation	-	-	miR-124	[58]
hsa_circ_0020256 (cir-NSMCE4A)	Tumor-associated macrophages	Up	Proliferation, migration, invasion	-	-	miR-25-3p, miR-145	[59]
hsa_circ_0043469 (cir-ERBB2)	Bile exosomes	Up	CCA progression, metastasis	-	-	miR-217, miR-331-3p	[60]
hsa_circ_0030998 (cir-LAMP1)	Tissue	Up	Postoperative recurrence rates	-	-	miR-198, miR-124-3p	[61]
hsa_circ_0003930 (cir-GGNBP2)	Tissue	Up	Shorter OS, shorter RFS after surgical resection	√	-	miR-200b, miR-145	[62]
hsa_circ_0059961 (cir-ITCH)	Tissue	Down	Positive correlation with survival time	√	-	miR-7, miR-214-3p	[63]
hsa_circ_0008621 (cir-HMGCS1)	Tissue	Up	Shorter survival time, higher recurrence rate	√	-	miR-140-3p, miR-361-3p	[64]
